# Intestinal tumors in neurofibromatosis 1 with special reference to fatal gastrointestinal stromal tumors (GIST)

**DOI:** 10.1002/mgg3.927

**Published:** 2019-08-09

**Authors:** Heli Ylä‐Outinen, Niina Loponen, Roope A. Kallionpää, Sirkku Peltonen, Juha Peltonen

**Affiliations:** ^1^ Department of Cell Biology and Anatomy University of Turku Turku Finland; ^2^ Department of Pulmonary Diseases Turku University Hospital Turku Finland; ^3^ Department of Dermatology Turku University Hospital Turku Finland

**Keywords:** bowel, cancer, death certificate, epidemiology, mortality

## Abstract

**Background:**

Type 1 neurofibromatosis (NF1) is a genetic tumor predisposing Rasopathy. NF1 patients have an increased risk for developing benign and malignant tumors, but the occurrence of intestinal tumors has not been investigated at the population level.

**Methods:**

In this retrospective register‐based total population study, diagnoses of gastrointestinal tract tumors were retrieved from the Finnish Care Register for Health Care for 1,410 NF1 patients and 14,030 reference persons. We also reviewed the death certificates of 232 NF1 patients who died during years 1987–2013, and specifically searched for diagnosis of gastrointestinal stromal tumor (GIST).

**Results:**

The register analysis revealed an increased overall hazard ratio (HR) of 2.6 (95% CI 1.9–3.6) for intestinal tumors in NF1 compared to general population. The highest HR of 15.6 (95% CI 6.9–35.1) was observed in the small intestine. The focused analysis of NF1 death certificates and GISTs demonstrated that the GIST was the primary cause of death in seven patients.

**Conclusion:**

This study emphasizes the need for careful evaluation of NF1 patients with gastrointestinal complaints. The challenge in diagnosis is that the tumors preferably occur at the small intestine, which is difficult target for diagnostic procedures. We also show that the NF1 GISTs may lead to fatal outcome despite of benign histopathological findings at the time of the diagnosis.

## 1 INTRODUCTION

Type 1 neurofibromatosis (NF1; OMIM 162200) is an autosomal and dominantly inherited cancer predisposition syndrome, caused by constitutional mutations in the *NF1* gene encoding the protein neurofibromin (Gutmann et al., 2017; Jouhilahti, Peltonen, Heape, & Peltonen, 2011; Uusitalo et al., 2016). Neurofibromin promotes the conversion of active Ras‐GTP to inactive Ras‐GDP and thus downregulates Ras downstream effectors including the mitogen activated protein kinase pathway (Gutmann et al., 2017; Korf, 2000; Wallace et al., 1990). Gastrointestinal stromal tumors (GISTs) are thought to originate from mesenchymal cells of Cajal (Al‐Shboul, 2013). However, the molecular pathogenesis may vary. In general population, most GISTs harbor an activating mutation in either the *KIT* encoding for receptor tyrosine kinase (Mast/stem cell growth factor) or the *PDGFRA* (platelet‐derived growth factor receptor alpha) (von Mehren & Joensuu, 2018). In NF1, the development of GISTs has been shown to result from the inactivation of both alleles of *NF1* gene (Maertens et al., 2006). Other pathogenic mechanisms of GISTs include mutations of succinate dehydrogenase complex subunits, or rare mutations in genes such as *BRAF, PIK3CA, CBL, or KRAS* (Gopie et al., 2018; Wada, Arai, Kure, Peng, & Naito, 2016).

An increased incidence of different types of gastrointestinal tract tumors has previously been described in NF1, mostly comprising of neuroendocrine neoplasms and GISTs (Agaimy, Vassos, & Croner, 2012; Garrouche et al., 2018). GISTs are estimated to occur in up to 6%–7% of NF1 patients (Nishida et al., 2016; Zöller, Rembeck, Odén, Samuelsson, & Angervall, 1997), although the diagnosis and registration of asymptomatic and small GISTs may be incomplete. The incidence of GISTs in NF1 patients has been estimated to be markedly higher compared to that of general population (Nishida et al., 2016; Søreide et al., 2016; Uusitalo et al., 2016). Typically, GISTs in NF1 patients manifest at middle age or later. NF1 patients tend to develop multiple GISTs, most often located in the small intestine, whereas half of cases in the general population occur in the stomach and are typically solitary (Wada et al., 2016). Most of NF1 GISTs are clinically indolent, with spindle cell type histology, with a low mitotic rate and good prognosis, but some GISTs can behave aggressively with fast and invasive growth and metastases typically seen in the liver and peritoneum (Andersson et al., 2005; Fletcher, Bridge, Hogendoorn, & Mertens, 2013; Miettinen & Lasota, 2013; Nishida et al., 2016).

Patients with GIST may be symptomless or may suffer from gastrointestinal discomfort, jaundice, weight loss, anemia, gastrointestinal bleeding, abdominal pain, palpable abdominal mass, or intestinal obstruction (Scarpa et al., 2008). The diagnosis of GIST in NF1 may be challenging because of the typical location of the tumor in the small intestine. The traditional diagnostic approach is based on colonic or gastroesophageal endoscopies which may fail to detect tumors of the small intestine. Other diagnostic tools include computed tomography (CT), magnetic resonance imaging (MRI), or positron emission tomography. CT or ultrasound‐guided needle biopsies or laparoscopy may be required (Garrouche et al., 2018; Nishida, Kawai, Yamaguchi, & Nishida, 2013).

The recommended primary therapy for all GISTs is surgical resection of the tumor regardless of its molecular pathology (Casali et al., 2018; Miettinen & Lasota, 2013; Poveda et al., 2017). Chemotherapy with tyrosine kinase inhibitor imatinib is generally recommended as a treatment of GISTs containing *KIT* or *PDGFRA* mutations (Casali et al., 2018; von Mehren & Joensuu, 2018). However, when GISTs are associated with NF1 or SDH syndromes, the treatment with imatinib is ineffective and not recommended (von Mehren & Joensuu, 2018). Several other newer tyrosine kinase and multikinase inhibitors have been used as a second line therapy to overcome imatinib resistance in *PDGFRA* or *KIT* mutated GISTs, but there are little data on multikinase inhibitors in NF1 GISTs. Some published reports have shown that some of NF1 GISTs may respond to sunitinib, an inhibitor of a broad spectrum of receptor tyrosine kinases (von Mehren & Joensuu, 2018; Mei, Du, Idowu, Mehren, & Boikos, 2018).

In this study, we performed a register‐based analysis of the hospital visits (1987–2014) of the 1,410 patients included in the Finnish NF1 cohort (Uusitalo et al., 2015) to explore the distribution of tumors in the gastrointestinal tract in NF1. We also reviewed all 232 death certificates of NF1 patients who died in Finland during years 1987–2013 in order to find cases where GIST was the primary or major contributing factor of death.

## 2 MATERIALS AND METHODS

The study adhered to the principles of the Declaration of Helsinki and was approved by the Ethics Committee of the Hospital District of Southwest Finland. Research permits were obtained from Finland's Ministry of Social Welfare and Health, the National Institute for Health and Welfare, Statistics Finland and all participating hospitals.

### 2.1 NF1 patients

The Finnish NF1 cohort was collected by searching all hospital visits with a diagnosis of NF1 in the 15 secondary and 5 tertiary referral centers of Finland during 1987–2011, as previously described (Uusitalo et al., 2015). The medical records of the patients were manually reviewed to ascertain NF1 diagnoses according to the National Institutes of Health diagnostic criteria (NIH consensus development conference statement, 1988).

### 2.2 Site‐specific analysis using data from the Finnish Care Register for Health Care

The Finnish Care Register for Health Care (HILMO; https://thl.fi/fi/web/thlfi-en/statistics/information-on-statistics/register-descriptions/care-register-for-health-care) is maintained by the National Institute for Health and Welfare and contains information on visits to specialized outpatient care and patients discharged from inpatient care. The register has information on inpatient care since 1969, while the outpatient visits have been registered since 1998.

Data from the Finnish Care Register for Health Care were available for 1,410 NF1 patients. In addition, a control cohort of 14,030 persons was formed by including 10 persons matched by sex, age, and municipality for each NF1 patient. The follow‐up of the patients with NF1 started at the first NF1‐related hospital visit during the ascertainment period (1987–2011), and the follow‐up of the controls started on the same day as the follow‐up of the respective NF1 patient. The follow‐up ended at the first occurrence of a diagnosis of interest, death, emigration, or end of follow‐up period on December 31st, 2014. The total follow‐up time was 21,220 person‐years among patients with NF1 and 229,314 person‐years among controls.

Diagnoses related to benign or malignant tumors of the gastrointestinal tract were retrieved for the NF1 patients and controls using the International Classification of Diseases (ICD) 9 and 10 diagnosis codes. The following codes were searched: ICD‐9:150–154, 211.0–211.4, 230.1–230.6; ICD‐10: C15‐21, D00.1‐D00.2, D01.0‐D01.3, D12.0‐D12.9, D13.0‐D13.3, D37.1‐D37.5, and D48.42. The diagnosis codes were grouped according to anatomical sites: esophagus, gastric region, small intestine, large intestine, rectum, and anus. ICD codes for the parenchymal organs in the abdomen, mesentery, and retroperitoneal space were excluded since the analysis was focused on intestinal tumors. An analysis combining diagnoses across the whole‐gastrointestinal tract was also performed. Persons with diagnoses related to several sites were included in the analyses of each separate site but only once in the analysis combining all sites. Hazard ratios were computed using the Cox proportional hazards model with frailty term to account for the matching of NF1 patients and controls. The data fulfilled the proportional hazards assumption of the Cox model. The analysis was conducted using the R software version 3.5.1 and package survival (version 2.42‐6).

### 2.3 Analysis of death certificates

The death certificates of deaths which occurred between January 1, 1987 and July 31, 2013 were retrieved for 1,453 NF1 patients (755 women, 698 men) from Statistics Finland. A total of 237 patients had died, and the complete death certificate was obtained for 232 patients (117 women, 115 men). The death certificates were searched for GIST. If the death certificate mentioned GIST, the medical history of the patient was studied in more detail using hospital records.

## 3 RESULTS

### 3.1 The location and incidence of gastrointestinal tract tumors in NF1

First, the Finnish Care Register for Health Care was searched for health care contacts associated with diagnoses of gastrointestinal tract tumors (Table 1). Since the data contain no information on the morphology of the tumors, the analysis encompasses all types of intestinal tumors including GISTs. The overall incidence of gastrointestinal tract tumors was higher in NF1 compared to the matched controls, the hazard ratio (HR) being 2.6 (Table 1). The highest HR, 15.6, was observed for tumors occurring in the small intestine, whereas the lowest HR of 1.2 was observed in the stomach.

**Table 1:** Hazard ratios of gastrointestinal tract tumors stratified by anatomical location in patients with NF1 compared to control population

Site	ICD−9 and ICD−10 codes	NF1 (*n*)	Controls (*n*)	HR	95% CI	*p* (NF1 vs. controls)
Esophagus	150^a^, 211.0, 230.1, C15^a^, D00.1, D13.0	4	8	5.7	1.7 to 18.9	.005
Stomach	151^a^, 211.1, 230.2, C16^a^, D00.2, D13.1, D37.1	4	37	1.2	0.4 to 3.4	.721
Small intestine	152^a^, 211.2, C17^a^, D13.2, D13.3, D37.2	14	10	15.6	6.9 to 35.1	<.001
Colon	153^a^, 211.3, 230.3, C18^a^, D01.0, D12.0‐D12.6, D37.3, D37.4, D48.42	24	151	1.9	1.2 to 2.9	.004
Rectum, rectosigmoid junction, and anus	154^a^, 211.4, 230.4–230.6, C19, C20^a^, C21^a^, D01.1‐D01.3, D12.7‐D12.9, D37.5	13	69	2.2	1.2 to 4.1	.008
All combined		52	240	2.6	1.9 to 3.6	<.001

Abbreviations: CI, confidence interval; HR, hazard ratio; ICD, International Classification of Diseases; *n*, number of patients; NF1, neurofibromatosis type 1.

a Including all subcategories.

### 3.2 Analysis of death certificates for GISTs in NF1 patients

Analysis of the death certificates of 232 NF1 patients revealed eight certificates which contained the diagnosis of GIST. The cause of death was GIST in seven patients, whereas one patient died because of malignant peripheral nerve sheath tumor (MPNST). Three of the patients were men and five were women. Hospital records of all eight NF1 GIST patients were available for study and the information on patients’ clinical characteristics and features of GISTs were collected retrospectively from the records (Table 2).

**Table 2:** Clinical, histopathological, and molecular characteristics of GISTs of eight patients with NF1

Patient	Location at the time of diagnosis	Tumor size/ multiple tumors	Morphology	Metastases at the time of diagnosis	Mitoses^a^ or Ki67%	Antibody CD117	Antibody CD34	Mutation analyses
1	Jejunum	11 cm/ yes	Spindle, areas of necrosis, and hemorrhage	Mesenterium	10%–15%	+	++	*KIT ‐* *PDGFRA ‐*
2	Ileum	20 cm/ yes	Spindle, areas of necrosis, and hemorrhage	No	10%	±	++	*N*/A
3	Duodenum	3.5 cm/ yes	Mixed spindle and epithelioid	No	1%	++	+	*N*/A
4	Jejunum	20 cm/ no	Spindle, areas of necrosis, and hemorrhage	No	>5	+	±	*KIT ‐* *PDGFRA ‐*
5^b^	Retroperitoneal	8.8 cm/ yes	Spindle, areas of necrosis, and hemorrhage	Liver Kidney	>5	−	*N*/A	*N*/A
6^c^	Ileum Jejunum	10 cm/ yes	Spindle	Mesenterium	>5	±	*N*/A	*KIT ‐* *PDGFRA ‐*
7	Duodenum	1.5 cm/ yes	Spindle	Mesenterium	<5 0%	+	*N*/A	*N*/A
8	Jejunum	6 cm/ no	Mixed spindle and epithelioid	No	>5 20%	±	++	*KIT ‐* *PDGFRA ‐*

Abbreviations: GIST, gastrointestinal stromal tumors; N/A, not available; NF1, neurofibromatosis type 1.

a Mitoses per 50 high‐power fields (area of 5 mm^2^).

b Earlier ileocecal GIST operated 27 years before the current GIST.

c Earlier retroperitoneal GIST operated 11 years before the current GIST.

The age of NF1 patients at the diagnosis of GIST varied from 35 to 74 years, and the median diagnosis age was 61 years (58 years in women and 65 in men). All patients were symptomatic at the time of diagnosis. The symptoms described in the hospital records were abdominal pain, anemia, palpable mass, weight loss, or nausea. The NF1 GIST was located most commonly in the small intestine (Table 2). In one patient, GIST was located in the retroperitoneal space. The histological appearance was the spindle cell type in most cases, but two GISTs were mixed spindle and epithelioid type (Table 2). GISTs were multiple in six patients and solitary in two patients. The diameter of the tumors varied from 1.5 to 20 cm. Metastases were observed in four patients at the time of the GIST diagnosis. Two patients had an earlier history of GIST surgery, 11 and 27 years before the current and fatal GIST (Table 2).

The immunostaining for CD117 (c‐Kit) was positive in seven of NF1 GISTs and five tumors were positive for CD34. Other antigens commonly used for tumor characterization were S‐100, desmin, vimentin, actin, smooth muscle actin, cytokeratin, and DOG1 (data not shown). Only four GISTs were analyzed for mutations at the time of the diagnosis, and all were negative for *KIT* and *PDGFRA* mutations. At the time of diagnosis, six of the eight GISTs were estimated to have a high risk for malignant behavior (size over >5 cm, and >5 mitoses per 50 high‐power microscopic fields, or Ki67 >5%), whereas the risk was low in two patients suggesting benign prognosis (Li et al., 2018; von Mehren & Joensuu, 2018; Segales‐Rojas, Lino‐Silva, Aguilar‐Cruz, & Salcedo‐Hernández, 2018; Zhou et al., 2017). However, the two NF1 patients with the low‐risk primary tumors in the duodenal localization died of GIST within one year of diagnosis.

The first treatment was surgery for all eight patients, followed by chemotherapy in five patients. Postoperative sequential chemotherapy lines continued until tumor progression. The first‐line chemotherapy was imatinib in all five patients, followed by sunitinib in two patients, and finally followed by subsequent paclitaxel and sorafenib in one patient. The survival time after the diagnosis of GIST varied from 1 day to 2 years 6 months. The median time from tumor detection to death was 6 months among the seven NF1 patients with a fatal GIST.

## 4 DISCUSSION

In order to focus on the burden of gastrointestinal tract tumors in NF1, we performed a site‐specific analysis using the Finnish Care Register for Health Care. The analysis revealed that the gastrointestinal tract tumors were significantly more common among NF1 patients than among the control persons. The tumors in the small intestine showed especially high hazard ratio of 15.6 (*p* < .001). The analysis was based on nationwide register data on patients discharged from inpatient care and visits to specialized outpatient care using the site‐specific ICD‐9 and ICD‐10 diagnosis codes. Although the register‐based data do not allow dissecting the exact tumor types, the high incidence observed in the small intestine may largely be due to GISTs that are known to typically occur in the small intestine in NF1 (Andersson et al., 2005). The analysis highlights and strengthens the understanding that the NF1 syndrome predisposes to tumors of the gastrointestinal tract.

We have previously reported an increased risk of GIST in NF1 with a standardized incidence ratio of 34.2 using register data from the Finnish Cancer Registry (Uusitalo et al., 2016). However, the Finnish Cancer Registry does not cover GISTs completely, since most GISTs are benign tumors and are not coded as cancers. Because GISTs can be located in different parts of intestine, a site‐specific search using ICD coding cannot identify them. In order to identify fatal GISTs occurring in NF1 patients, we analyzed all 232 death certificates of the patients in the Finnish NF1 registry (Uusitalo et al., 2015). Furthermore, we studied the medical records of the patients with GIST mentioned in the death certificates. We regret that the current data do not allow the comparison between fatal and non‐fatal GISTs since identifying clinical characteristics specifically associated with fatal GISTs in NF1 would be clinically significant.

Death certificates revealed eight NF1 patients with GIST. Seven of them died of GIST, whereas one patient died of MPNST. All eight had symptoms typical for GIST, such as abdominal pain, anemia, palpable mass, weight loss, or nausea. Most of the GISTs had spindle cell morphology, they were multiple and often located in the small intestine, concordant with previous literature (Nishida et al., 2016). The original risk estimate for intestinal GISTs predicted high risk for malignant behavior in six tumors and benign behavior in two. Five of the six high‐risk NF1 patients ultimately died of GIST. On the other hand, the prognostic scheme failed to predict the outcome in two NF1 patients whose primary tumors were located in the duodenum and were originally classified as low‐risk tumor. It remains to be seen if the duodenal GISTs in NF1 are prone to more malignant behavior than initially estimated based on current criteria (Hong et al., 2018). Surgery was the primary intervention in the eight patients. Chemotherapy with imatinib was given to five patients, followed by other multikinase inhibitors in two patients. No responses to chemotherapy were observed. The insensitivity to chemotherapy is in line with the earlier reported lack of the mutations in the *PDGFRA* or *KIT* genes (Mussi, Schildhaus, Gronchi, Wardelmann, & Hohenberger, 2008).

In conclusion, morbidity related to different gastrointestinal tract tumors is high in NF1 patients. The most challenging site for the diagnosis of different tumors and specifically GISTs is the small intestine, since endoscopy may be insufficient. We suggest that middle aged or older NF1 patients with gastrointestinal symptoms should be further examined with an abdominal CT or MRI scan. Our findings also suggest that NF1 patients should be carefully followed after tumor excision, even if the risk estimate for malignancy is low, because the risk estimates may not be fully applicable to NF1 GISTs. Our results emphasize the fact that GISTs in NF1 are not always indolent but may lead to death.

## CONFLICT OF INTEREST

None declared.

5

## Data Availability

The Finnish National Institute for Health and Welfare provides data for researchers who meet criteria. The application for authorization can be done at https://www.thl.fi/en/web/thlfi-en/statistics/information-for-researchers/authorisation-application. Data from NF1 database may be provided for researchers who meet the criteria to access confidential data.
